# Contextualising clinical reasoning within the clinical swallow evaluation: A scoping review and expert consultation

**DOI:** 10.4102/sajcd.v68i1.832

**Published:** 2021-07-29

**Authors:** Thiani Pillay, Mershen Pillay

**Affiliations:** 1 Discipline of Speech-Language Pathology, School of Health Sciences, University of KwaZulu-Natal, Durban, South Africa; 2 Speech and Language Therapy, Massey University, Auckland, New Zealand; 3 Department of Health Professions, Manchester Metropolitan University, Manchester, United Kingdom

**Keywords:** clinical reasoning, dysphagia, clinical swallow evaluation, speech-language pathology, contextualisation

## Abstract

**Background:**

This study explored the available literature on the phenomenon of clinical reasoning and described its influence on the clinical swallow evaluation. By exploring the relationship between clinical reasoning and the clinical swallow evaluation, it is possible to modernise the approach to dysphagia assessment.

**Objectives:**

This study aimed to contextualise the available literature on clinical reasoning and the CSE to low-middle income contexts through the use of a scoping review and expert consultation.

**Method:**

A scoping review was performed based on the PRISMA-ScR framework. The data was analysed using thematic analysis. Articles were considered if they discussed the clinical swallow evaluation and clinical reasoning, and were published in the last 49 years.

**Results:**

Through rigorous electronic and manual searching, 12 articles were identified. This review made an argument for the value of clinical reasoning within the clinical swallow evaluation. The results of the study revealed three core themes related to the acquisition, variability and positive impact of clinical reasoning in the clinical swallow evaluation.

**Conclusion:**

The results of this review showed that the clinical swallow evaluation is a complex process with significant levels of variability usually linked to the impact of context. This demonstrates that in order to deliver effective and relevant services, despite challenging conditions, healthcare practitioners must depend on clinical reasoning to make appropriate modifications to the assessment process that considers these salient factors.

## Introduction

Clinical reasoning is a decision-making process that is vital to clinical practice (Higgs & Jones, 2008; Young et al., 2020). It has been understood as a complex and critical skill (Doeltgen, Attrill, & Murray, 2019; Findyartini, Hawthorne, McColl, & Chiavaroli, 2016; Miles, Friary, Jackson, Sekula, & Braakhuis, 2016), which allows healthcare practitioners (HCPs) to provide services in a flexible manner that embraces uncertainty within the clinical encounter (Greenhalgh, Wherton, Shaw, & Morrison 2020). This is of particular importance to low-middle income contexts (LMICs), such as South Africa, where flexible and responsive reasoning is required to provide equitable and relevant healthcare services (Sachs, 2012; Saito et al., 2016). The value of clinical reasoning is well established within dysphagia practice. Doeltgen et al. (2019), and many others (Jones, Cartwright, Whitworth, & Cocks, 2018; Mathers-Schmidt & Kurlinski, 2003; McAllister et al., 2020; Pettigrew & O’Toole, 2007; Rumbach, Coombes, & Doeltgen, 2018), have shown that sound clinical reasoning is vital within dysphagia assessment and management to prevent poor patient outcomes. However, almost all this research has emerged from higher income contexts which cannot always be generalised to LMICs.

The aim of our article, therefore, was to explore the nature of clinical reasoning within the clinical swallow evaluation (CSE) in published literature over the last 49 years using a scoping review and expert consultation. This was achieved through two study objectives which included: (1) determining how clinical reasoning had been characterised within the CSE regarding its explicitness, conceptual understanding, reported perceptions and perceived value in literature and, (2) determining the relevance of the scoping review findings to policy and practice in a South African context.

## Theoretical background

The theoretical background of this article rationalises and deconstructs the use of clinical reasoning within practice. Clinical reasoning is a complex cognitive process used by HCPs to decide on appropriate diagnoses and treatments for patients (Gummesson, Sundén, & Fex, 2018; Miles et al., 2016; Young et al., 2020). This involves the use of cognitive tools like interpretation, association and evaluation to critically consider presenting clinical information to inform management plans (Gummesson et al., 2018; Young et al., 2020). Levett-Jones et al. (2010) describe clinical reasoning as a recursive and cyclic process to collect appropriate cues, establish goals and execute actions in an appropriate manner. Clinical reasoning was initially hypothesised as a static skill which was applied verbatim to every situation (Gruppen, 2017). However, Márquez-Álvarez, Calvo-Arenillas, Talavera-Valverde and Moruno-Millares (2019), Young et al. (2020) and McBee et al. (2018), have suggested the importance of accounting for the individualised nature of clinical reasoning which considers the patient, context, evidence and clinician in decision-making. This is valuable as it allows HCPs to consider multiple sources of information when developing a working hypothesis regarding patients (Boshuizen et al., 2018). In this article, we focus on the effect of clinician, context and assessment modality on clinical reasoning.

### Clinician

Clinical reasoning is highly dependent on the practicing clinician (Jessee, 2018; Langridge, Roberts, & Pope, 2016). Cognition, training and experience are unique to each clinician, but are all influential within the clinical reasoning process (Norman et al., 2017). The role of these factors have informed various clinical reasoning models within the literature (Norman et al., 2017), that are broadly classified within two categories. The *subconscious* and *conscious* (Gummesson et al., 2018) models of clinical reasoning are discussed below.

The *subconscious* or pattern-recognition model assumes clinical reasoning is influenced by knowledge acquired from experience and intuition (Johansen & O’Brien, 2016). This model is informed by the premise that HCPs associate clinical problems presenting in current patients with problems seen previously and thus manages patients in a similar manner (Benner, Hughes, & Sutphen, 2008; Johansen & O’Brien, 2016). According to this model, novice clinicians initially rely on training and established guidelines to make clinical decisions. Progressively, they move towards intuitive reasoning as they become more experienced (Benner & Tanner, 1987; Jessee, 2018). In contrast, the *conscious* model, the hypothetico-deductive model, states that HCPs develop a hypothesis, based clinical information which is informed by training and experience (Abdallah, 2020; Trimble & Hamilton, 2016). This hypothesis is then exhaustively tested through several stages such as cue recognition, hypothesis generation, cue interpretation and hypothesis evaluation, to develop a clear management strategy (Abdallah, 2020; Trimble & Hamilton, 2016).

### Context

Clinical reasoning is determined by various factors related to context and political consciousness (Kathard & Pillay, 2013; Young et al., 2020). This is particularly true for LMICs all over the world, who are haunted by political and economic injustices (Browne et al., 2016; Lavoie et al., 2018; Umeh & Feeley, 2017; Ziltener & Künzler, 2013). These injustices have resulted in several consequences such as severe inequity, high unemployment rates and insufficient health systems (Baatiema et al., 2020; Browne et al., 2016; Ruelas, Gómez-Dantés, Leatherman, Fortune, & Gay-Molina, 2012). This in turn has caused unsurmountable personnel and resource constraints, high patient numbers, as well as a complex burden of disease (Ludwick, Morgan, Kane, Kelaher, & McPake, 2020; Mayosi et al., 2012; Meng et al., 2020; Puchalski Ritchie et al., 2016). These constraints have forced HCPs to practice in a creative and flexible manner to combat the effect of these inequities on their patients (Baatiema et al., 2020; Saito et al., 2016; Van Graan, Williams, & Koen, 2016). For perceived non-essential services such as speech therapy, these difficulties are often compounded because of their lack of priority when disseminating resources (Andrews & Pillay, 2017; Bright, Wallace, & Kuper, 2018; Rech, Hugo, Schmidt, Goulart, & Hilgert, 2019). As a result, according to Andrews and Pillay (2017), Pierpoint and Pillay (2020) and Modi and Ross (2000), many Speech-Language Therapists (SLTs) chose to alter their practice patterns to cope with the demands of the contexts in which they work, to allow for best possible practice within challenging conditions (Doeltgen et al., 2019; Saito et al., 2016).

According to Kathard and Pillay (2013), another facet of contextual influence is determined by political climates and historical biases. This accounts for how political, social, cultural and systemic structures impact clinical reasoning (Kathard & Pillay, 2013). For example, HCP’s clinical reasoning is informed by knowledge acquired from professional training (Doeltgen et al., 2019). Historically, academic instruction has subscribed to a privileged approach to knowledge which has been taught under the guise of universality (Dawson, 2020; Grosfoguel, 2013; Kathard & Pillay, 2013). This is problematic as a rigid approach to knowledge which gives deference to colonial ideals cannot account for the complex social, economic and historical realities of the rest of the world (Dawson, 2020; Ndlovu-Gatsheni, 2018). Therefore, it is important for us to understand the influence of these biases on clinical reasoning to assist in managing patients in a manner that is more relevant and socio-politically aware (Findyartini et al., 2016; Kathard & Pillay, 2013; Zahir, Miles, Hand, & Ward, 2020).

### Assessment

In this article, we have chosen to foreground the CSE given its subjective nature which relies on clinical reasoning (Garand, McCullough, Crary, Arvedson, & Dodrill, 2020). The CSE is an assessment which evaluates patients to make inferences about the presence, nature and cause of dysphagia within the oral and pharyngeal phase of the swallow (Carnaby-Mann & Lenius, 2008; Garand, McCullough, Crary, Arvedson, & Dodrill, 2020). This is achieved through chart reviews, patient observations, oral motor examinations and multiple swallow trials (Doeltgen et al., 2019; Garand et al., 2020). The CSE is a detailed assessment which informs hypotheses regarding the anatomy and physiology of the swallow (Rangarathnam & McCullough, 2016). This assessment is necessary as it allows SLTs to make decisions regarding oral intake and consistency, additional compensations and ultimately the need for instrumental assessments (Namasivayam-MacDonald & Riquelme, 2019; Virvidaki, Nasios, Kosmidou, Giannopoulos, & Milionis, 2018). There is significant controversy regarding the utility of the CSE as a stand-alone assessment to guide dysphagia management (Doeltgen, McAllister, Murray, Ward, & Pretz, 2018; Riquelme, 2015), given its reliance on perceptual information and varied practice patterns (Riquelme, 2015). However, the CSE is still the most commonly used form of dysphagia assessment (Virvidaki et al., 2018). It is regarded as a time efficient, cost-effective and non-invasive assessment, which allows therapists to understand a patient’s dysphagia in relation to their medical history and displayed symptoms (Carnaby-Mann & Lenius, 2008; Riquelme, 2015; Virvidaki et al., 2018).

## Methods

This study followed a scoping review design which aimed to collate and understand the evidence available on clinical reasoning (Aromataris & Munn, 2017). The Preferred Reporting Items for Systematic Reviews and Meta-analyses Extension for Scoping Reviews (PRISMA-ScR) (Tricco et al., 2018), framework was adapted to guide the study. Although the PRISMA-ScR is a novel framework, the clarity it provided for methodological quality and reporting styles made it appropriate to ensure a transparent and complete review could be carried out (Chang, 2018; Mueller et al., 2013). The 22-item checklist (Appendix 1) was adapted for the needs of the study. Given the nature of our second objective, we acknowledge that the methodology employed differs from traditional approaches to scoping reviews. However, this adapted methodology was instrumental to contextualising the findings to a South African context to account for global narratives on the utility of clinical reasoning.

This study consisted of three phases namely: data preparation, collection and analysis. As preparation for the main study, the first phase involved a pilot study to test the developed research instruments (i.e. search protocols, screening protocols and tools) to establish internal validity (Kinchin, Ismail, & Edwards, 2018). Additionally, an expert reference group consisting of five dysphagia academics in South Africa were consulted as per the Joanna Briggs Institution (JBI) (Peters MDJ, Godfrey C, McInerney, Munn, Tricco & Khalil, 2020) to assist in informing decisions regarding keywords, databases, search terms and strategies for searching the literature. The second phase involved the collection and review of the literature identified from various sources. Peer-reviewed articles and grey literature published between 1970 and 2019 were considered. The researchers focused on the last 49 years as research published prior was considered outdated. Within the third and final phase of the study, the data was analysed and a focus group was consulted to contextualise the results as per objective two.

### Eligibility criteria

The studies included were those which:

- Were published between 1970 and 2019.
- Assessed the swallowing function of human participants.
- Were published by dysphagia practitioners in medically related fields (e.g. speech-pathology, dietetics, dentistry).
- Evaluated the assessment of patients using the CSE.
- Referred to clinical reasoning or its associated terms.

### Information sources

Information for this review was sourced from both electronic and physical journals. The electronic databases searched were: South African Bibliographical Network (SABINET), EBSCOhost, Medline, PubMed, Clinical Key, Google Scholar and Cochrane Library. These databases were selected as they are highly reputable and extensive, which ensured that all the relevant literature was identified. Furthermore, published conference papers of American Speech and Hearing Association (ASHA), and South African Association of Speech, Language and Hearing association (SASLHA), as well as postgraduate dissertations available on *ResearchSpace* were all searched to identify grey literature. The final electronic search was conducted on 03 June 2019.

Handsearching was performed at three South African university libraries. The Biomedical Journal, Academic Medicine, Medical Education, South African Journal of Communication Disorders, International Journal of Speech and Language, New England Journal of Medicine, and Journal of Medical Research were searched from 1970 to 2019 for keywords via table of contents and subject indexes. The final manual search was conducted on 07 June 2019.

### Search strategy

The JBI’s (Peters et al., 2020), three-step search strategy was utilised to guide a comprehensive search of the literature. This included searching two databases namely, Google Scholar and MEDLINE, for articles which related clinical reasoning to the CSE to identify keywords contained within the title, abstract, and index (Moola et al. 2015). The keywords identified through this strategy were: clinical reasoning, clinical decision-making, CSE, dysphagia and speech therapy. Following this, a second search was carried out to determine which combination of these keywords would yield the most relevant results across databases. The final combination of keywords used were: [clinical reasoning] OR [decision-making] AND [dysphagia assessment] OR [swallow assessment]. These terms were placed into the databases using advanced searching options to filter articles by date and search terms. Finally, reference lists of relevant articles were searched post data collection to ensure no relevant information was missed.

### Study selection

Two reviewers blindly screened the titles, abstracts and full articles of the search results independently. Reviewer 1 performed the manual search and screening, and Reviewer 2 searched and reviewed the six electronic databases. Upon completion of the screening processes, the reviewers met to discuss the eligibility of each article for the dataset. The suitability of each of the articles was evaluated based on the inclusion criteria. An agreement rate of 85.7% was achieved between the reviewers. In the two instances where the reviewers could not reach a consensus, a third reviewer was consulted to make the final decision.

### Data charting and analysis

The data extraction and charting process was iterative, allowing for the dataset to evolve as useful elements were identified (Moola et al., 2015; Schultz et al., 2018). The data charted included: author, year of publication, country, aims, study population and size, methodology, intervention type, outcomes and key findings. The extracted data served as a basic summary to help orient readers to the nature of the research collected.

Thematic analysis as described by Braun and Clarke (2006), was utilised to provide a rich and complex account of the data. Braun and Clarke (2006), described a six-step recursive method for thematic analysis which included: (1) familiarisation, (2) generation of initial codes, (3) searching for and (4) reviewing themes, (5) production of final themes, and (6) a report. Furthermore, the computer analysis software NVivo 12 was used to support data coding processes by streamlining complex functions like syntactical word recognition, word frequency counting, coding and the creation of visual representations of the data in the form of word clouds, tree diagrams and word matrices.

### Consultation

Finally, the researcher chose to include an optional consultation as described by Colquhoun et al. (2017), within the third phase of this review. Although largely unknown, the consultation phase is a historic (Arksey & O’Malley, 2005; Levac, Colquhoun, & O’Brien, 2010) and current (Faulkner, Taylor, Ferrence, Munro, & Selby, 2006; Faulkner et al., 2011; Khan et al., 2015; Moore et al., 2019; Myers, Schaefer, & Coudron, 2017; Smartt et al., 2019; Youssef, Chaudhary, Wiljer, Mylopoulos, & Sockalingam, 2019; Zwiep et al., 2018) feature of scoping reviews which engages stakeholders to understand the implications of a review’s findings within practice. The aim of this consultation phase was to contextualise the data to South Africa. The reliability, value and applicability of the results were addressed within the context of healthcare in South Africa. Four dysphagia practitioners were consulted through a focus group to allow for a multidimensional discussion around the collected data (Nyumba, Wilson, Derrick, & Mukherjee, 2018). The focus group was conducted using open-ended questions surrounding the applicability of the results to the South African context. Upon completion, the focus group was transcribed and thematically analysed in relation to the themes of the literature search. This allowed the researchers to highlight the applicability of the results in the context of South Africa.

### Ethical considerations

This study was approved by the Biomedical Research Ethics Committee (BREC) of the University of KwaZulu-Natal (BREC Reference number: BE275/19).

## Results

### Search results

**FIGURE 1:**
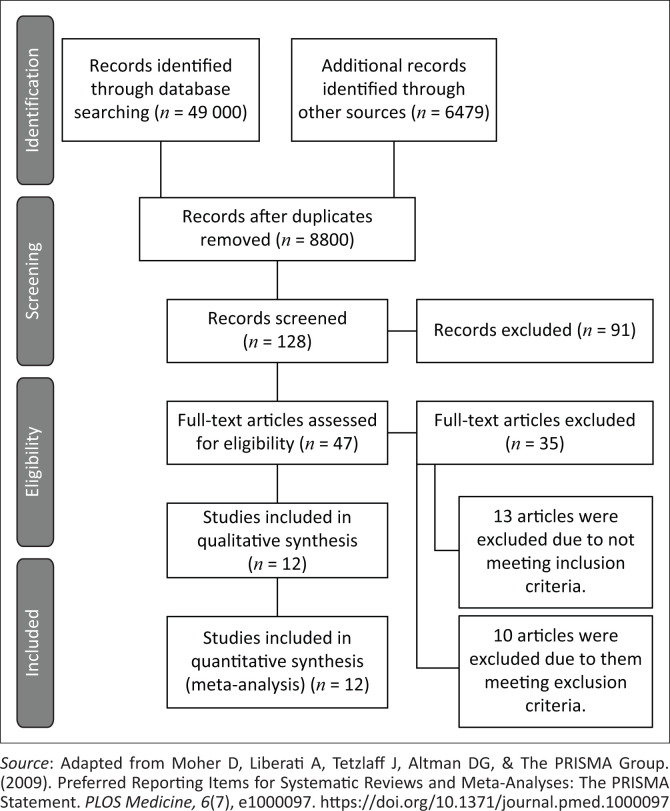
PRISMA-ScR flow diagram of results.

Figure 1 depicts the data collected through both the electronic and manual searches. There were 49,000 articles identified in the initial searches across the six databases, however there were extensive numbers of duplicates between the searches. After the duplicate studies were removed, 8,800 studies were screened. Of the total number of articles screened by title, only approximately 0.14% of the articles were included within the final review. This could demonstrate a poor focus within the literature on clinical reasoning within the CSE.

### Characteristics of the included studies

Appendix 2 represents the characteristics of the 12 studies included in the study. Each of the selected articles made explicit references to the use of clinical reasoning within the CSE as per the eligibility criteria. The studies were published between the period 1999 and 2019. The methodologies of the study included: two literature reviews, seven descriptive survey-based studies, two intervention studies and one case study. All the included studies were conducted in largely urbanised, high-income countries. Four studies were carried out in Australia, two in Ireland, four in the United States of America, one in Canada, and one in England. This influenced the narratives which could be explored given the homogenous economic context of the dataset (see Appendix 2).

### Thematic results

The results of the review revealed three main themes related to the value and use of clinical reasoning within the CSE. The results of both the review and the consultation highlighted several key issues including: the importance of clinical reasoning, the impact of training and experience, the importance of individualised service delivery and the value of flexible and critical reasoning within dysphagia assessment. Each of these themes will be elaborated on further in the discussion below.

## Discussion

The results of the literature search and consultation have been presented and discussed simultaneously. By presenting the results together, the researchers aimed to represent perspectives from both high-income and low-income contexts on clinical reasoning which correlates with the objectives of the study. Through the thematic analysis, three core themes regarding clinical reasoning in the CSE were revealed and will be elaborated in the following subsections.

### Theme 1: Experience matters in clinical reasoning

The review highlighted the importance of knowledge acquired from training and experience in the clinical reasoning process. As expected, university training was described as influential in the development of clinical reasoning skills. As mentioned previously, training is the foundation on which all clinical knowledge is built and therefore impacts how all decisions are made (Doeltgen et al., 2019). According to seven of the articles within the dataset, a solid theoretical understanding of assessment and management of dysphagia is needed to develop appropriate knowledge schemas to guide reasoning (Brodsky, Mayfield, & Gross, 2019; Doeltgen et al., 2018; Jones et al., 2018; Mathers-Schmidt & Kurlinski, 2003; McAllister, Kruger, Doeltgen, & Tyler-Boltrek, 2016; McCurtin & Carter, 2015; McCurtin & Healy, 2017).

However, an interesting finding was the focus on experience rather than training as the guiding factor within clinical reasoning. Several articles within the dataset referred to the level and quality of experience amongst SLTs as being the preferred factor to guide clinical reasoning (Brodsky et al., 2019; Doeltgen et al., 2018; Jones et al., 2018; Mathers-Schmidt & Kurlinski, 2003; McAllister et al., 2016; McCurtin & Carter, 2015; McCurtin & Healy, 2017; Pettigrew & O’Toole, 2007; Rumbach et al., 2018; Walshe, Ryan, & Regan, 2017). Table 1 demonstrates this with several extracts from the dataset which support the use and preference of experience in clinical reasoning. Experience was described in terms of the Recognition-Primed Decision Model (RPDM), which theorises that HCPs make decisions through the recognition of patterns (Klein, 1999). All 12 articles described pattern recognition as useful, as it allows HCPs to quickly process information to develop a small number of plausible diagnoses. This preference for pattern-recognition was attributed to it being a resource- and time-efficient process whilst avoiding haphazard or inaccurate decisions. The RPDM relies heavily on intuition, which is described as ‘the immediate realisation of risk, usually learnt […] without conscious awareness’ (Farr-Wharton, Brunetto, & Shacklock, 2012, p. 1392). According to articles 1 (Doeltgen et al., 2018), 7 (McCurtin & Carter, 2015) and 11 (McCurtin & Healy, 2017), intuition is developed through diverse and high-quality clinical experiences. Article 1 (Doeltgen et al., 2018) explains that these experiences help SLTs to develop complex networks of illness scripts and patterns of symptoms that are relied on to guide decision-making. Furthermore, articles 1 (Doeltgen et al., 2018) and 7 (McCurtin & Carter, 2015) highlighted that the amount of experience which HCPs have, impacts on both the speed and accuracy of their pattern-recognition approach. Novice HCPs have less information within their knowledge schemas to develop diagnoses at an intuitive level. Therefore, they have to engage in an exhaustive *conscious* approach to make diagnoses (Klein, 1999).

**TABLE 1:** Extracts regarding the value of clinical experience.

Article	Extracts
(Doeltgen et al., 2018)	‘High quality experiences are essential for developing accurate intuitive clinical judgments’.
(McCullough, Wertz, Rosenbek, & Dinneen, 1999)	‘Clinicians believe a number of bedside methods and measures that have no research support for their use should be included in a bedside examination. The absence of research support does not mean the procedure is of no value. It simply means that clinicians are relying on their experience’.
(McCurtin & Healy, 2017)	‘There is evidence within the discipline to suggest that practice evidence – a clinician’s own experience, colleagues’ experience and perhaps the discipline’s culture – plays a large part in framing practice’s decisions’.
(Pettigrew & O’Toole, 2007)	‘It is reasonable to propose that clinicians with more training/experience are more confident in their own skills and abilities and therefore less likely to rely on instrumental measurements’.
(McCurtin & Carter, 2015)	‘That for most day-to-day clinical situations; the evidence supporting decision making is experiential knowledge. Tonelli (2010) states that effectively clinical experience offers a way to help bridge the gap between research and care of the individual’.

**FIGURE 2:**
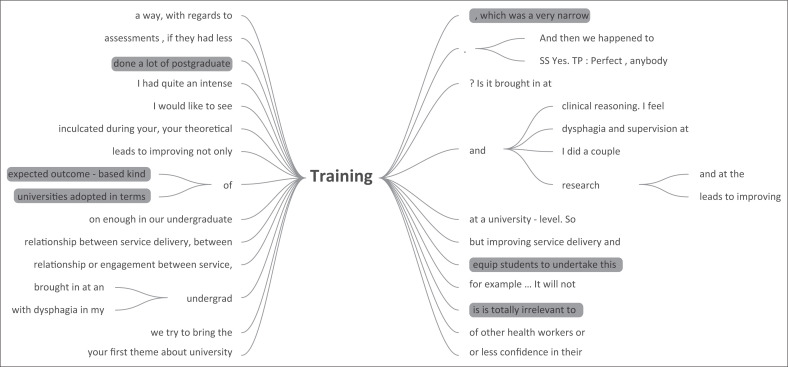
Word tree showing the conceptualisation of training in consultation phase by focus group, generated in NVivo 12.

The sentiment that experience is arguably more influential than training was echoed in the results of the consultation phase. This was attributed to inconsistency and irrelevance of training of SLTs at tertiary institutions. The results showed that practitioners preferred to rely on their own experiences to guide their approach to dysphagia management. As seen in Figure 2, the focus group felt that universities focused more on a theoretical understanding of dysphagia and trained clinicians in a linear outcome-based model which is inappropriate to the LMIC contexts that they find themselves in. They reported that this inappropriateness of training was exacerbated by insufficiency in opportunities to develop these clinical skills in real-life scenarios. These sentiments are echoed in recent literature which describes the doubt and under-preparedness voiced by many novice SLTs in response to their training (Caesar & Kitila, 2020; Coutts, 2019; Singh et al., 2015).

### Theme 2: Clinical reasoning cannot be decontextualised

**FIGURE 3:**
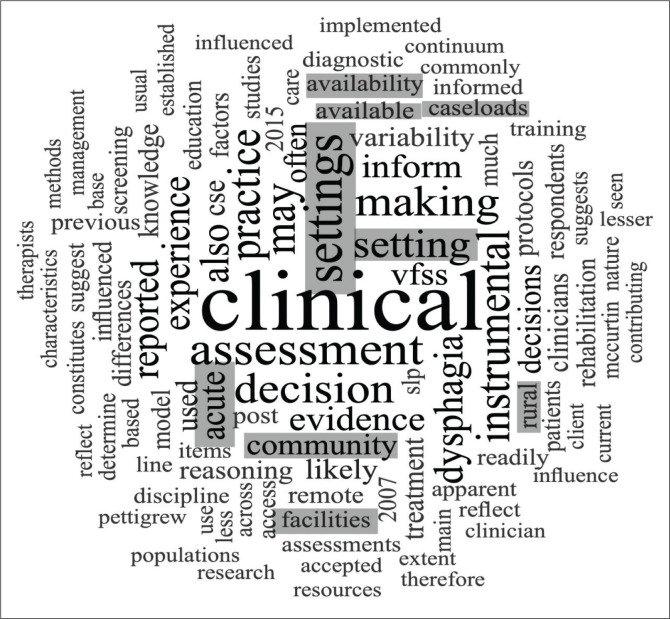
Word cloud of clinical setting, generated in NVivo 12.

**FIGURE 4:**
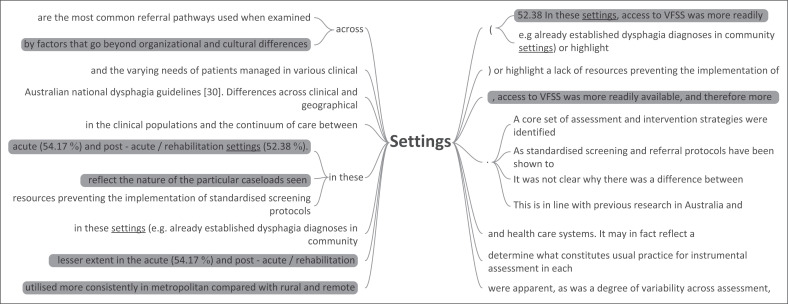
Word tree representing setting data, generated in NVivo 12.

Globally, SLTs often work in a variety of settings as seen in Figure 3. As a result of this diversity in setting, every article within the dataset highlighted contextual factors as a major contributing factor to clinical reasoning which should not be discounted. Article 7 describes this by stating: ‘the clinical decisions [*which*] health professionals [*make*] are strongly influenced by the context in which they are made’ ( McCurtin & Carter, 2015, p. 72). This makes sense given that context often determines resource availability, prioritised health goals, institutional policies and popularised assessment practices (Schwarz,Coccetti, & Cardell, 2020). As a result of this increased variability, HCPs are required to engage in assessment in a highly nuanced and complex manner. Therefore, we can deduce that the decision which clinicians make cannot be removed from the context in which it occurs. For example, the data showed a discrepancy between acute/subacute and community-based settings as demonstrated in Figure 4. SLTs favoured instrumental assessments within the acute/subacute settings. This higher frequency can be attributed to equipment usually being more accessible as well as the complex nature of the patient population. Conversely, SLTs in community settings were more likely to rely on the CSE to guide their decisions as instrumental assessments are considered costly and difficult to access. This is in line with the literature explored earlier by Virvidaki et al. (2018) and Namasivayam-MacDonald and Riquelme (2019).

The results of the consultation frequently described how influential context was in making clinical decisions. The clinicians felt that clinical reasoning was imperative to ensure that the management of patients reflected both the needs of the population as well as best practice even in challenging conditions. There was consensus that because of the complex quadruple burden of disease, severe resource constraints and high patient loads which are mirrored in most LMICs, SLTs have to alter the way in which they practice to cope with demands of their settings (Andrews & Pillay, 2017). This was described to be unique to LMICs as often there are fewer confounding variables to consider within high income contexts. Therefore, they felt that SLTs often relied on clinical reasoning when developing context-specific assessments which are crucial in effective and equitable service delivery.

### Theme 3: Focused clinical reasoning positively impacts patient outcomes

The dataset (as seen in Table 2) showed that SLTs have moved to a purposeful and process-driven approach to assessment which allows them to easily adapt to conditions. Articles 1 (Doeltgen et al., 2018), 4 (Jones et al., 2018), 7 ( McCurtin & Carter, 2015) and 12 (Martino , Pron, & Diamant, 2004) suggested that this has occurred because SLTs have a network of core assessment components which are influenced by a hierarchical model. According to this hierarchy, SLTs begin with the most common CSE components and then move on to less utilised components as required. This process continues until the clinician has developed a satisfactory diagnosis. González-Fernández, Ottenstein, Atanelov and Christian (2013), corroborated this idea by stating that component omission by SLTs was purposeful and non-random. This suggests that component selection in the CSE is reflective of the practice philosophy amongst SLTs who ‘[do] what works best for their patients, in their setting’ (Jones et al., 2018, p. 73). In addition, it is clear to see the positive impact which clinical reasoning has on the utilisation of the CSE. Within complex cases better outcomes are reported when SLTs employ high-quality clinical reasoning processes to guide the selection of assessment components. This conclusion is supported by Odderson, Keaton and McKenna (1995), who state that:

[*R*]egardless of the variability in SLT CSE practice, it has been demonstrated that SLT involvement in the clinical management of dysphagia indeed contributes to better clinical outcomes and reduced healthcare expenditure. (p. 1132)

**TABLE 2:** Data extracts on the impact of clinical reasoning.

Article	Quote
(Doeltgen et al., 2018)	‘The effectiveness of this global judgment is likely to arise from the SLP’s reasoning and decision making driving a deliberate combination of certain assessment items during the CSE to make a determination regarding the overall presentation of the patient’.
(McAllister et al., 2016)	‘Evidence that SLPs contribute positively to dysphagic patient outcomes [*3*] indicates that this clinical reasoning and decision-making process may be a sounder approach than following a strict item-based protocol or checklist. This would be in accordance with research on medical clinical reasoning that has found that diagnostic reasoning is not a linear process and is influenced by experience’.
	‘Although it is not yet clear what this clinical reasoning process is, it is well accepted and indeed recommended, that the patient and context should determine the assessment process [*6*] and the CBSA does support better outcomes for patients [*3*]’.
(McCurtin & Carter, 2015)	‘Higgs, Burn and Jones (2001), [*showed*] that clinicians frequently make decisions where there are no right and wrong solutions or actions [*using effective clinical reasonin*g]’.
(Rumbach et al., 2018)	Emerging evidence now suggests that this variability may reflect clinicians’ clinical reasoning processes and may be non-random.

The positive impact of clinical reasoning includes assisting SLTs in guiding management decisions, considering multiple factors, preventing unnecessary assessment and synthesising information from multiple sources.

This positive impact was extrapolated within the results of the consultation phase. The focus group agreed that there was substantive value in clinical reasoning especially within the complex socio-economic context of South Africa as an example of an LMIC. The participants reasoned that clinical reasoning was a key tool which clinicians used to adapt dysphagia assessment protocols to make them relevant to their context. They felt that this study was invaluable in creating a link between research, service delivery and training to meet the needs of the complex and diverse communities within LMICs in an effective manner.

## Limitations

There were limitations to this study, given the nature of the methodology and the time-sensitive and resource-sensitive nature of data collection. Firstly, the inclusion criteria were narrow to fit the scope of the research question which excluded studies that considered instrumental assessments or dysphagia management. However, given that the CSE is so dependent on clinical reasoning skills, it was valuable to foreground this modality. Secondly, the researchers acknowledge limitations regarding the implementation of the scoping review methodology. For example, the choice of search terms, databases and the nature of handsearching may have resulted in some relevant articles not being identified. However, the rigorous and transparent nature of the methodology has aided in mitigating these issues. Furthermore, for the consultation phase the researchers chose to use South Africa as the example of a LMIC for convenience. Whilst this could limit the applicability of the results, South Africa is considered an effective illuminator of the impact of wealth disparity within a country. Therefore, these results could be mirrored within many contexts globally.

## Conclusion

**FIGURE 5:**
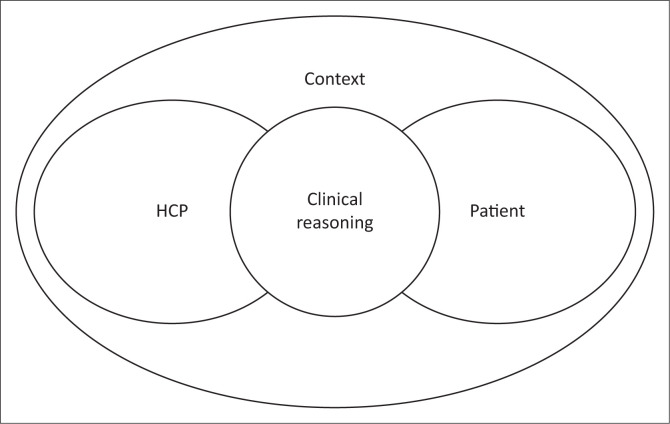
Venn diagram representing clinical reasoning.

In conclusion, this study demonstrated that the CSE is a highly complex process with significant levels of variability. This variability is evident because dysphagia treatment and management cannot be decontextualised. To deliver effective and relevant services despite challenging and complex conditions, HCPs must depend on clinical reasoning as a key tool to make appropriate decisions within the assessment process. Figure 5 is suggested to *re-present* the way in which we consider clinical reasoning based on the result of this review. It aims to account for the complex interaction between both internal and external factors which impact clinical reasoning skills. This review has highlighted the impact of both the clinician and context on clinical reasoning, particularly within LMICs. So, how are these factors not sufficiently emphasised within practice? In the future, HCPs have to better account for the effect of these factors within their clinical reasoning to provide equitable and effective services.

## Appendix 1

**TABLE 1-A1:** Preferred Reporting Items for Systematic reviews and Meta-Analyses extension for Scoping Reviews (PRISMA-ScR) Checklist.

Section	Item	PRISMA-ScR checklist item	Reported On page #
**Title**			
Title	1	Identify the report as a scoping review.	1
**Abstract**			
Structured summary	2	Provide a structured summary that includes (as applicable): background, objectives, eligibility criteria, sources of evidence, charting methods, results, and conclusions that relate to the review questions and objectives.	1
**Introduction**			
Rationale	3	Describe the rationale for the review in the context of what is already known. Explain why the review questions/objectives lend themselves to a scoping review approach.	1
Objectives	4	Provide an explicit statement of the questions and objectives being addressed with reference to their key elements (e.g. population or participants, concepts, and context) or other relevant key elements used to conceptualise the review questions and/or objectives.	1
**Methods**			
Protocol and registration	5	Indicate whether a review protocol exists; state if and where it can be accessed (e.g. a Web address); and if available, provide registration information, including the registration number.	N/A
Eligibility criteria	6	Specify characteristics of the sources of evidence used as eligibility criteria (e.g. years considered, language, and publication status), and provide a rationale.	3
Information sources†	7	Describe all information sources in the search (e.g. databases with dates of coverage and contact with authors to identify additional sources), as well as the date the most recent search was executed.	3
Search	8	Present the full electronic search strategy for at least 1 database, including any limits used, such that it could be repeated.	3
Selection of sources of evidence‡	9	State the process for selecting sources of evidence (i.e. screening and eligibility) included in the scoping review.	4
Data charting process¶	10	Describe the methods of charting data from the included sources of evidence (e.g. calibrated forms or forms that have been tested by the team before their use, and whether data charting was done independently or in duplicate) and any processes for obtaining and confirming data from investigators.	4
Data items	11	List and define all variables for which data were sought and any assumptions and simplifications made.	5
Critical appraisal of individual sources of evidence§	12	If done, provide a rationale for conducting a critical appraisal of included sources of evidence; describe the methods used and how this information was used in any data synthesis (if appropriate).	N/A
Synthesis of results	13	Describe the methods of handling and summarising the data that were charted.	5
**Results**			
Selection of sources of evidence	14	Give numbers of sources of evidence screened, assessed for eligibility, and included in the review, with reasons for exclusions at each stage, ideally using a flow diagram.	5
Characteristics of sources of evidence	15	For each source of evidence, present characteristics for which data were charted and provide the citations.	Appendix 2
Critical appraisal within sources of evidence	16	If done, present data on critical appraisal of included sources of evidence (see item 12).	N/A
Results of individual sources of evidence	17	For each included source of evidence, present the relevant data that were charted that relate to the review questions and objectives.	5
Synthesis of results	18	Summarise and/or present the charting results as they relate to the review questions and objectives.	5
**Discussion**			
Summary of evidence	19	Summarise the main results (including an overview of concepts, themes, and types of evidence available), link to the review questions and objectives, and consider the relevance to key groups.	5–7
Limitations	20	Discuss the limitations of the scoping review process.	8
Conclusions	21	Provide a general interpretation of the results with respect to the review questions and objectives, as well as potential implications and/or next steps.	8
**Funding**			
Funding	22	Describe sources of funding for the included sources of evidence, as well as sources of funding for the scoping review. Describe the role of the funders of the scoping review.	8

*Source:* Tricco, A.C., Lillie, E., Zarin, W., O’Brien, K. K., Colquhoun, H., Levac, D., … Weeks, L. (2018). PRISMA extension for scoping reviews (PRISMA-ScR): Checklist and explanation. *Annals of Internal medicine, 169*(7), 467–473. https://doi.org/10.7326/M18-0850

JBI, Joanna Briggs Institute; PRISMA-ScR, Preferred Reporting Items for Systematic reviews and Meta-Analyses extension for Scoping Reviews; N/A, not applicable.

† , Where *sources of evidence* (see second footnote) are compiled from, such as bibliographic databases, social media platforms, and Web sites.

‡ , A more inclusive/heterogeneous term used to account for the different types of evidence or data sources (e.g. quantitative and/or qualitative research, expert opinion, and policy documents) that may be eligible in a scoping review as opposed to only studies. This is not to be confused with *information sources* (see first footnote).

¶ , The frameworks by Arksey and O’Malley (6) and Levac and colleagues (7) and the JBI guidance (4, 5) refer to the process of data extraction in a scoping review as data charting.

§ , The process of systematically examining research evidence to assess its validity, results, and relevance before using it to inform a decision. This term is used for items 12 and 19 instead of ‘risk of bias’ (which is more applicable to systematic reviews of interventions) to include and acknowledge the various sources of evidence that may be used in a scoping review (e.g. quantitative and/or qualitative research, expert opinion, and policy document).

## Appendix 2

**TABLE 1-A2:** Characteristics of included studies.

Data set #	Author and Date	Population studied	Total participants	Study design	Country and income level	Aims of the study
1	Doeltgen, McAllister, Murray, Ward, and Pretz (2018)	Not applicable	Not applicable	Review of the literature	Australia (high income)	Overview the relevant literature pertaining to reasoning and decision-making in the CSE
2	McAllister et al. (2016)	Speech-language pathologists	308	Intervention based study	Australia (high income)	Understanding clinical decision-making for component selection by SLTs in the CSE
3	Rumbach, Coombes, and Doeltgen (2018)	Speech-language pathologists	154	Survey-based questionnaire analysed descriptively	Australia (high income)	Understanding practice patterns in Australia based SLTs in the CSE
4	Jones, Cartwright, Whitworth, and Cocks (2018)	Speech-language pathologists	118	Survey-based questionnaire analysed descriptively	Australia (high income)	Investigating the practices of SLTs in Australia in treatment post stroke
5	Felix, Joseph, and Daniels (2019)	Speech-language pathologists	1	Case-study based review	United States of America (high income)	Highlights the considerations in the evaluation and management of dysphagia
6	Pettigrew and O’Toole (2007)	Speech-language pathologists	70	Survey-based questionnaire analysed descriptively	Ireland (high income)	Investigating the dysphagia evaluation practices in Irish SLTs
7	McCurtin and Carter (2015)	Speech-language pathologists	271	Mixed methods; online survey-based questionnaire and focus groups	England (high income)	Explore professional knowledge and clinical decision-making in SLT practice
8	Brodsky, Mayfield, and Gross (2019)	Not applicable	Not applicable	Review of available literature/Seminar of literature	United States of America (high income)	Review of literature pertaining to the CSE in ICU environments
9	Mathers-Schmidt, and Kurlinski (2003)	Speech-language pathologists	64	Survey-based questionnaire analysed descriptively	United States of America (high income)	Determine the nature of evaluation and decision-making within the CSE amongst SLTs
10	McCullough, Wertz, Rosenbek, and Dinneen (1999)	Speech-language pathologists	61	Survey-based questionnaire analysed descriptively	United States of America (high income)	Determine which components are commonly used to assess swallowing
11	McCurtin and Healy (2017)	Speech-language pathologists	116	Survey-based questionnaire analysed descriptively	Ireland (high income)	Understanding why/how SLTs chose the components in the CSE
12	Martino, Pron, and Diamant (2004)	Speech-language pathologists	23	Test-retest survey design	Canada (high income)	Understanding practice patterns in SLTs
