# Corrigendum: Contextualising clinical reasoning within the clinical swallow evaluation: A scoping review and expert consultation

**DOI:** 10.4102/sajcd.v70i1.873

**Published:** 2023-09-08

**Authors:** Thiani Pillay, Mershen Pillay

**Affiliations:** 1 Discipline of Speech-Language Pathology, School of Health Sciences, University of KwaZulu-Natal, Durban, South Africa; 2 Speech and Language Therapy, Massey University, Auckland, New Zealand; 3 Department of Health Professions, Manchester Metropolitan University, Manchester, United Kingdom

In the published article, Pillay, T., & Pillay, M. (2021). Contextualising clinical reasoning within the clinical swallow evaluation: A scoping review and expert consultation. *South African Journal of Communication Disorders, 68*(1), a832. https://doi.org/10.4102/sajcd.v68i1.832, there was a mistake in Figure 1 as published. The number of records excluded should be 81 rather than 91. The corrected Figure 1 appears as follows.

**The original incorrect:**

**FIGURE 1:**
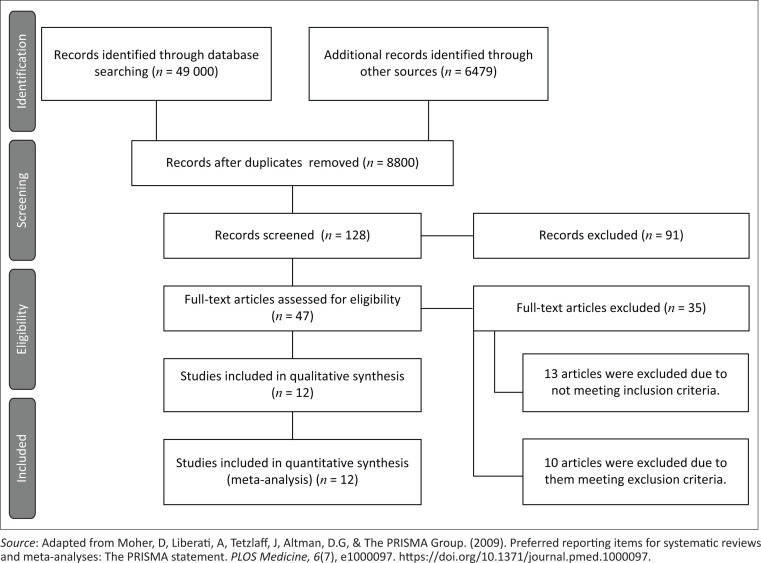
PRISMA-ScR flow diagram of results.

**The revised and updated:**

**FIGURE 1:**
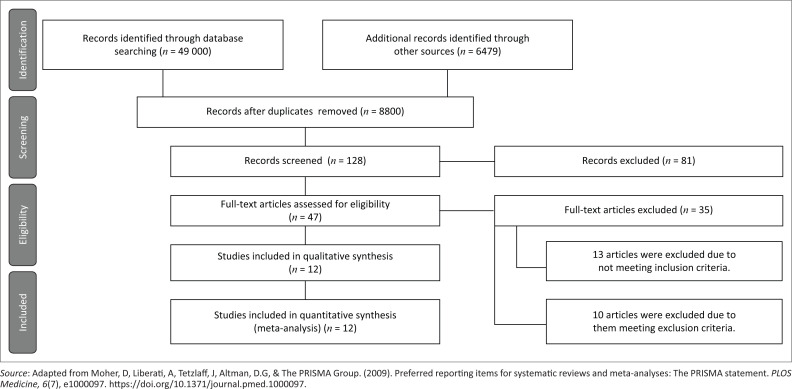
PRISMA-ScR flow diagram of results.

The authors apologise for this error. The correction does not change the significance of study’s findings or overall interpretation of the its results or the scientific conclusions of the article in any way.

